# Organ failure as an expression of organ remodeling. Involvement of oxidative stress

**Published:** 2013-09-25

**Authors:** VG Dincă, G Manole

**Affiliations:** Preclinical Studies Department, “Titu Maiorescu" University of Bucharest

**Keywords:** organ failure, apoptosis, necrosis, fibrosis, extracellular matrix

## Abstract

Organ failure can be defined as a relevant concept of functional-anatomical condition of body structural change. Anatomical, functional disorders of the body are the expression of structural remodeling. The process is complex, including the first functional cell mass reduction (in particular, apoptosis and necrosis), and on the other hand the development of fibrosis process with a starting point at the extracellular matrix. Both mechanisms that take place are caused by etiological factors, enhanced, favoring the developer cytokines mediated by inflammatory processes such as premature death and functional constituent cells of the body; secondly the inflammation leads to fibrosis. The article review is a summary of the knowledge that exist at the level of the anatomical organ remodeling.

## Introduction

Expressing serious disturbance in the function of the clinical plan of the anatomical organ failure translates the existence of a remodeling/ reshuffled architectonics organ expression conduct of at least two fundamental processes: 

 - Activation of functional cell death by apoptosis induced necrosis, or by similar processes; 

 - Replacing damaged cells in the interstitial connective tissue, as a result to this hyperplasia [**1**]. 

 Progressive organ failure is the result of a dynamic process, which allows the recognition of the stages of induction and development, potentially reversible up to a point in disease progression. In the first step, after the damage caused by the etiologic agent, the installation of protoplasmic pH changes as an expression of the tendency to develop metabolic acidosis by altering cellular metabolic processes. The initiation and development of various morphological and functional disturbances in apoptotic cells are stimulated by the generation of the etiologic agent of the disease, but back activation of cytokines, produced either by stimulated cells in organ structure or by local migrated cells, which generally belong phagocyte system mononuclear. In a later stage, cytokines, through their autocrine, paracrine or metacrine action, stimulate the release of enzymes and proteolytic cell-destructive acts primarily as caspases and/ or dominant effect at the gap, as metalloproteinases (MMPs = matrix metalloproteinases) [**2**]. The main actions developed by the active substances (cytokines and chemokines) which are released are designed to increase vascular permeability, cell adhesion and migration, to be recruited to lesion sites, cellular infiltrate (polymorphonuclear, mononuclear system cells - macrophages) [**3**]. 

 First, the degranulation recruits monocytes, bossing them, and then they are marginalized, so that the diapedesis and migration to reach the lesion focus chimiotactism and/ or electro-attraction. As a result of binding to apoptotic cells stimulated by TSP, monocytes become macrophages engaged in phagocytosis in the area of inflammation. At the launch, the purpose of phagocytosis is only to remove necrotic cells and those that have been partially destroyed, without the intention of developing inflammation. However, an inflammatory response occurs, leading to the initiation of the remodeling architectural conditions of the body, due to the capacity of the mononuclear cell free system, the local active functional products and/ or enzymes involved in inflammation. They will join those released from damaged cells of the body, empowering and mutually action [**4**]. 

 The remodeling is focused in particular on the activation (proliferation and differentiation), fibroblasts are accomplished by the action developed by pro-inflammatory cytokines, such as PAI-1 (Plasminogen Activator Inhibitor-1), osteopontin, growth factors and prostaglandins. In parallel, some of them, such as PAI-1 exercise and control role, intervening matrix remodeling extracellular [**5**]. 

 Destructive lesions cell activate as the level of extracellular matrix fibrosis recognizing a common cytotoxic mechanism, developed through self-healing by oxidative stress [**6**].The theory admits oxidative stress as a basic principle, excessive growth ROS7 production [**7,8**]. 

 The main cell types that can be incriminated as being responsible for the development of oxidative stress organ level are: 

 - Cells stimulated incorporation of pro-apoptotic body; 

 - Fibroblasts, myofibroblasts and any of the epithelial and/ or endothelial cells at the same level; 

 - Cells migrated as neutrophils and monocytes - macrophages. 

 The common property of these cells is their ability to respond to stimulation-induced activation of the etiologic agent, resulting in the production and release of at least three classes of active compounds: 

 a. cytokine with autocrine and paracrine action, such as growth factors or cytokines pro-generating fibrosis; 

 b. proteolytic enzymes, acting primarily in the extracellular matrix, such as MMPs [**9**]; c. angiogenic factors like angiotensin II (Ag II); 

 The common characteristic of these factors is that the acts associated release, while stimulating and empowering themselves, self-sustaining deteriorative anatomic and functional processes of the body, the body reshaping. Functionally, the process becomes one fix clearing destructive one, because beyond cell death induced etiology, cellular inflammatory mechanisms, immune and last but not least, ischemic destruction occurs [**4**]. Ischemia occurs as the ultimate mechanism of fibrosis and fibrogenesis that compresses arteriolar lumen stenosis is produced, and path deviates by an excess quantity of collagen, where other proteins of the extracellular matrix are deposited. In such cases, ischemia is responsible for the imbalance between tissue blood flow and metabolic consumption, externalized by inducing alterations apparently free of new cells and discharge of proteolytic enzymes, activating local inflammatory and immune mechanisms, developer-fibrotic destructive effects; self-sustaining process [**1**]. 

 Anatomically, it was recognized that the optimum performance of the functions of an organ assumes a proportional development not only in architectural and structural terms - functional cells but also of the intercellular matrix. The latter development reflects a balance between the production and the degradation of collagen; homeostasis is permanently regulated particularly on the synthesis process. Understanding the mechanisms of fibrogenesis is facilitated by defining its biochemical characteristics as the synthesis of extracellular matrix proteins in the basal membrane and/ or interstitium [**10**]. The way it was defined made fibrosis be still unclear, the results of experimental studies arguing on a direct anabolic fibroblast exercised by various active compounds synthesized locally or systemically. 

 The original etiologic factor generating organ damage induces pro-apoptotic stimulation only structural-functional cells of the body, and later, through various mechanisms, inflammatory-immune and exercising ischemic-proliferative and destructive action of free cells mobilized for defense. The same mechanisms have chemotactic and mitogenic functions of endothelial cells around, favoring the formation of neo-vessels as they migrate towards the center of the area of lesion fibrosis. 

 The development process is conditioned by the apoptotic fibrosis, at its turn dependent on the balance of a variety of protein molecules pro-and anti-apoptotic. 

 The timing of the initiation and production disturbances induced apoptosis cell death is able to determine the action expression developed by extracellular stimuli/ intracellular apoptotic message bearers, responsible for modulating the activity of various enzymes involved in cell death. 

 When the organ extracellular matrix is remodeled, the fibrosis process can generate 2 models of fibrosis: diffuse or localized. If diffuse fibrosis is secondary to the excessive accumulation of collagen fibers in the intercellular spaces, the focus can be located perivascularly, increasing ischemia by increasing the distance crossed by the arteriolar oxygen to the cells. For example, myocardial fibrosis, generating failure, an excessive deposit of collagen proteins can be produced around cardiomyocytes - endomysium community and around groups of cardiomyocytes - perimisium community. 

 As an expression of exacerbation processes of proliferation and diversification of fibroblast function, fibrosis is modulated by both local cytokines and by a number of hormones, synthesized locally and systemically, such as aldosterone and angiotensin II (Ag II). 

 By binding to specific receptors, hormones, minerals and corticosteroids are involved not only in excessive production of ROS in vascular development, tissue inflammation, but also in the activation of fibrosis [**11-13**]. Moreover, the actions developed through-corticoid receptor stimulation minerals and which reinforce each other, and several which developed by increasing oxidative stress on both apoptotic cells and on fibroblasts stimulated the injured body [**13**]. These intricate actions developed while clearly demonstrating the myocardium, allowing the use of antagonist minerals and hope-corticosteroid therapy in various forms of congestive heart failure, including those that occur after the myocardial infarction. 

 The results of experimental studies admit that Ag II acts in developing the two-stage fibrosis: 

 A. In the first stage, the active angiotensin induces increased cytokine osteopontin expression, the protein responsible for the organization and stability of extracellular matrix due to its ability to bind to the various gap structures: collagen, fibronectin etc. [**14-16**]. 

 B. In the second stage, the active angiotensin potentiates the pro-fibrotic developed by minerals and hormones, steroids, acting as a factor stimulating growth factors which regulate the expression and synthesis of collagen [**16,17**]. 

 In order to develop cellular effects, both hormones mentioned, just as various pro-fibrotic cytokines serve as ligands to cell membrane receptors and communicate with the nucleus and/ or cytoplasm of the cell by activating pathways in cytoplasmic signal carrier. Most of these pathways that transmit such signals are used as transducer protein Ras proteins attached to the internal face of the cellular membrane [**18-20**]. Due to the coupling of various ligands, Ras subfamily proteins specific membrane receptors are activated. In this form - Ras-GTP - acts as a transducer that initiates various cellular processes, including apoptosis. Although it is now widely accepted that inflammation precedes fibrosis, experimental studies have shown that fibrosis is not always in control of inflammation. As far as stimulation is concerned, the Ras protein induction develops and action/ development of inflammation and fibrosis is under the direct control of MMPs and their tissue inhibitors (TIMP = Tissue Inhibitors of Matrix metalloproteinases) [**21,22**]. Functionally, in terms of normal tissue metabolism, MMPs are inactive and in conditions of hypoxia-ischemia, oxidative stress or other factors, such as the Lezion action, are activated. The results of the experimental studies on animals have shown that the excess of MMPs leads to progressive tissue remodeling, while reducing the inhibition of MMPs. The duration and intensity of action developed by MMPs are modulated in at least three ways: the action of tissue inhibitors, the speed of the auto degradation and the endocytosis of selective [**23**]. 

 The action developed by MMPs in the extracellular matrix is dependent on the degree of quantitative expression of three of the four types of time because they have been identified to date, only isoforms: TIMP1, TIMP3 and TIMP4 and the fibroblasts. Both the activity of MMPs and TIMPs that is controlled by the intensity of their transcription, regulation of the quantity that can be modulated, synthesis of cytokines, such as: 

 - Tumor necrosis factor alpha (TNFα); 

 - Interleukins IL-1, IL-4, IL-6 etc. [**24**] 

 - Growth factors - transformation: EGF, HGF, TGF-ß, etc. The presence of TGF-β in the extracellular matrix is an indirect factor reduction of body cell mass, which is functional as it acts as an inhibitor of the cellular proliferation [**23**].

## Conclusions

The difficulty in understanding the mechanisms of organ failure resulting from complexity, which includes various pathogens, such as inflammation, oxidative stress, neuro-hormonal activation, damage (to varying degrees, by apoptosis) of cells that migrated from the body or the local purpose defense. 

The functioning at an optimum capacity of the cellular signal is conditioned by the existence of a constant quantity of agents ROS/ RNS, the activation of oxidative stress leads to an exacerbation of ROS signaling, which is responsible for the genesis of structural alterations at the cellular and interstitial level. 

 Although there have been important advances in the knowledge of the mechanisms by which induced cell death and organ fibrosis are installed, there are still many unknown aspects, which justify the increasing interest of research to identify them, in the hope that they will be able to discover new therapeutic means, turning an irreversible process into a reversible one.
